# Acupuncture for the treatment of the pain-fatigue-sleep disturbance-numbness/tingling symptom cluster in breast cancer survivors: a feasibility trial

**DOI:** 10.1007/s00520-024-08529-9

**Published:** 2024-05-07

**Authors:** Ki Kyung Kwon, Judith Lacey, Kim Kerin-Ayres, Gillian Heller, Suzanne Grant

**Affiliations:** 1 NICM Health Research Institute, Western Sydney University, Sydney, NSW Australia; 2 Chris O’Brien Lifehouse Hospital, Camperdown, NSW Australia; 3 University of Sydney, Camperdown, NSW Australia

**Keywords:** Acupuncture, Integrative oncology, Cancer, Symptom clusters, Model validity

## Abstract

**Purpose:**

Breast cancer survivors following disease-modifying treatment frequently experience multiple-concurrent symptoms (Jansana et al. in Int J Cancer 149(10):1755 1767, 2021), negatively impacting their quality of life and increasing the risk of polypharmacy (Alwhaibi et al. in J Oncol Pharm Pract 26(5):1052 1059, 2020). This study evaluates the feasibility and acceptability of acupuncture for the management of the pain-fatigue-sleep disturbance-numbness/tingling symptom cluster in breast cancer survivors, and investigates relationships between the symptom cluster and Traditional Chinese Medicine (TCM) syndrome diagnosis.

**Methods:**

This was a single-arm, pre-test/post-test feasibility trial conducted at Chris O’Brien Lifehouse Hospital, Australia. Breast cancer survivors who completed treatment and experienced clinically significant levels of two or more symptoms (pain, fatigue, sleep disturbance, numbness/tingling) were eligible to participate in the individualized, pragmatic 6-week acupuncture intervention. The primary outcome was feasibility and acceptability. Effectiveness was explored using a symptom cluster mean score.

**Results:**

Twenty women enrolled in the study over an 11-week period and 90% completed the study. Most women agreed or completely agreed that acupuncture was feasible (85%), acceptable (90%), and appropriate (90%). Both mean and composite symptom cluster scores were significantly reduced (*p* < 0.001), as were individual symptom scores in fatigue (*p* < 0.001), sleep disturbance (*p* = 0.04), and numbness/tingling (*p* = 0.01). TCM syndromes most closely associated with this symptom cluster were *Spleen qi deficiency* and *Heart fire*. No adverse events were reported.

**Conclusion:**

This study demonstrated that acupuncture was safe and feasible, justifying a powered randomized control trial. Preliminary findings suggest beneficial effects of acupuncture for the management of the pain-fatigue-sleep disturbance-numbness/tingling symptom cluster for women with breast cancer. TCM syndromes identified in this trial may be used to guide acupuncture treatment protocols.

Clinical trial registration: This trial was registered with the Australian New Zealand Clinical Trials Registry (ACTRN12622000590763) on 21 April 2022.

**Supplementary Information:**

The online version contains supplementary material available at 10.1007/s00520-024-08529-9.

## Introduction

Symptoms and side effects associated with breast cancer and its treatment rarely occur in isolation [1, 2]. Symptoms are often managed separately, and multiple interventions may be required for rehabilitation, with significant financial cost [3]. Globally, more than 2.1 million women are diagnosed with breast cancer annually [4] and 83.7% of women surviving 5 years and more experience multiple morbidities [5]. The “one intervention for one symptom” approach has led to an increased dependence on polypharmacy for breast cancer survivors (50–74%), where older women with breast cancer are prescribed an average of seven medications [6, 7]. Polypharmacy is associated with frailty, reduced function, risk of falls, and mortality, exposing the limitations of managing single symptoms independently in the presence of multiple-concurrent symptoms [8].

Symptoms experienced by breast cancer survivors can be inter-related, sharing a common mechanism or a pathway, occurring as a “symptom cluster” [9, 10]. A symptom cluster is a group of two or more symptoms that have a pattern of occurring concurrently [9]. A symptom cluster management approach gathers multiple-concurrent symptoms into a single cluster that can be managed simultaneously [10]. Potential benefits include reducing the need for multiple interventions, reducing financial cost, reducing the risk of polypharmacy and its associated risks, and improving quality of life in cancer survivorship [2, 11].

Fatigue, pain, sleep disturbance, and numbness/tingling are among the most common symptoms in breast cancer survivors [12]. These symptoms are often identified together [13], with a “pain-fatigue-sleep” cluster being reported in 40–80% of the general cancer population [14] and a “pain-fatigue-psychological” cluster being reported in 13% of the breast cancer population [15]. Symptoms in the pain-fatigue-sleep disturbance-numbness/tingling cluster were frequently reported in a study of patients accessing acupuncture services at a major cancer hospital in Sydney [16].

To date, there is no single pharmacological therapy available to treat symptom clusters, and many of these individual symptoms remain difficult to treat. There is rising interest in non-pharmacological integrative approaches in onocology such as exercise, mindfullness, and yoga [17, 18], with the Society of Integrative Oncology (SIO) and American Society of Clinical Oncology (ASCO) recommending evidence-based integrative approaches in the management of specific symptoms [19]. Both Schnyer and Hullender Rubin [20] and National Cancer Institute [21] recommend exploring symptom clusters as outcomes.

Acupuncture, using Traditional Chinese Medicine (TCM) syndrome diagnosis, may provide an option for symptom cluster management. Using a TCM approach, patient’s signs and symptoms are considered together as a “TCM syndrome,” a concept similar to a clinical phenotype (a particular pattern of multiple-concomitant symptoms), rather than single-independent symptoms [22]. In traditional clinical practice, the TCM syndrome diagnosis guides the treatment prescription, with acupuncture points, needle stimulation, depth, and retention selected to best treat that syndrome [23]. The symptom cluster management approach and TCM approach to patient care are similar, both considering the relationship between a group of inter-related/concurrent symptoms.

Acupuncture has demonstrated benefit in a range of single symptoms in women with breast cancer [24]. Acupuncture is recommended for general cancer-related pain and aromatase-inhibitor-related arthralgia in ASCO-SIO guidelines [25]. Emerging evidence suggests that acupuncture may also lead to reductions in chemotherapy-related peripheral neuropathy (CIPN) presented as “numbness and tingling” [26], cancer-related fatigue (CRF) [27], and sleep disturbance [28]. To our knowledge, there have been no clinical trials investigating acupuncture for symptom clusters using a TCM approach to date.

The primary aim of this study was to examine the feasibility of acupuncture for the pain-fatigue-sleep disturbance-numbness/tingling symptom cluster in women with breast cancer who have completed treatment. The secondary aim was to investigate the correlation between TCM syndromes and this symptom cluster and monitor the change in prevalence and burden of the symptom cluster over the course of the intervention.

## Methods

### Design

The study was a pragmatic, single-arm, single-site, feasibility trial conducted at a major cancer hospital, Chris O’Brien Lifehouse Hospital (COBLH), in Sydney, Australia. This trial was prospectively registered (ACTRN12622000590763) and ethical approval obtained from St. Vincent’s Human Research Ethics Committee, Sydney (2021/ETH00152). The study is reported according to the CONsolidated Standards of Reporting Trials (CONSORT) statement for feasibility trials and the STandards for Reporting Interventions in Clinical Trials of Acupuncture (STRICTA) guidelines (Supplementary material 1).

### Recruitment and screening

Women were recruited using flyers, a QR code within the hospital, and through referral from hospital doctors and therapists. Screening was completed through a short REDCap questionnaire or by phone. Women were provided with a copy of the participant information sheet prior to obtaining written informed consent. Reasons for exclusion, declining, and withdrawals were documented.

### Study participants

To be eligible, women with breast cancer over the age of 18, who had completed primary disease-modifying treatment (breast cancer survivors), had to report a minimum of two of the following symptoms:

- Pain, fatigue, sleep disturbance, and/or numbness/tingling and
- Two or more of these symptoms were rated as ≥ 4 out of 10 using the Edmonton Symptom Assessment Scale 17 (ESAS-17). A score of 4 or more is considered a clinically significant symptom burden [29, 30].

Potential participants were not eligible for the trial if they had recurrent or metastatic cancer, were receiving cancer-modifying treatment such as chemotherapy or radiotherapy (except those being treated with hormone therapy, or Trastuzumab and/or Pertuzumab), pregnant at the time of enrolment, had thrombocytopenia (defined as equal to or less platelet count of 80), or had received acupuncture in the last 2 weeks (potential participants were allowed a 2-week washout period). Participants who had demand-type pacemakers in situ could participate in the trial, but were excluded from receiving electroacupuncture.

### Intervention

Participants received eight acupuncture treatments over 6 weeks (twice per week for the first 2 weeks, and once per week for the ensuing 4 weeks). The study intervention was designed to reflect clinical practice, with two experienced and credentialed oncology acupuncturists developing individualized treatment protocols based on symptom presentation and syndrome differentiation. TCM syndrome diagnosis based on TCM theory and clinician expertise guided point selection [31, 32]. Syndrome diagnosis was documented using Sub-Optimal Health-50 questionnaire (SOHQ-50).

Between 10 and 15 needles were used per treatment. Needles were manipulated to activate *de-qi* sensation (physiological response to the needle such as numbness, pressure, heaviness) [31] and retained for 20–30 min. Vinco brand, stainless steel, and filiform acupuncture needles were used, with sizes varying from 0.16 × 0.20 to 0.25 × 0.40. Needle insertion depth varied from point to point, based on traditional recommendations [31].

Vinco brand earseeds were used at the acupuncturist’s discretion. Participants were instructed to leave the earseed in situ until the next treatment or disposed of if they fell off. Electroacupuncture was used at the discretion of the acupuncturist.

Acupuncturists providing the treatments were trained in Australia, registered with the Australian Health Practitioners Regulation Agency (AHPRA), and had a minimum of 5-year experience working with people with cancer.

### Outcome measures

All patient report outcome measures were collected from participants using an electronic link on REDCap. Acupuncturists were blinded to these assessments with the exception of the ESAS-17 and the TCM questionnaire (Sub-Optimal Health Questionnaire-50). The ESAS-17 and the TCM questionnaires were used to guide clinical treatment. The outcome assessor was not involved in the treatment process. Assessments were made at baseline, end of treatment (week 6), and at follow-up (week 8) (Supplementary material 2).

### Primary outcome

The primary outcome was the feasibility of acupuncture as management of the symptom cluster, measured by the following:

1. The Acceptability of Intervention Measure (AIM), Intervention Appropriateness Measure (IAM), and Feasibility of Intervention Measure (FIM). These four item questionnaires are validated measures for assessing the acceptability, appropriateness, and feasibility of an intervention [33].
2. Recruitment rate (number of enrolments / number of eligible participants), retention rate (number that completed the end of treatment questionnaire / number enrolled), adherence rate (number completing six or more treatments / number enrolled), compliance rate (number of appointments that were attended / number of total appointments), and follow-up rate (number that completed follow-up questionnaire / number enrolled) [34]. Feasible rates were defined as recruitment of 20 participants over 16 weeks, 75% retention, and compliance rate.

### Secondary outcomes

We also sought a signal on effectiveness through changes from baseline in the symptom burden of the pain-fatigue-sleep disturbance-numbness/tingling cluster using the modified Edmonton Symptom Assessment Scale-17 (ESAS-17) and quality of life using the Patient Reported Outcome Measure Information System-29 (PROMIS-29) [30, 35]. A mean “symptom cluster” score that could be compared to other single symptoms was calculated.

To understand any correlations between the pain-fatigue-sleep disturbance-numbness/tingling symptom cluster and TCM syndrome diagnosis, we used the Sub-Optimal Health Questionnaire-50. See Supplementary material 3 for further details.

All minor and major adverse events were recorded using the Common Terminology Criteria for Adverse Events (CTCAE).

### Sample size

Twenty women were recruited for the intervention arm, based on previous studies that recommend a sample of 12 to 30 participants in a group for feasibility trials [36, 37].

### Statistical methods

Statistical analysis was conducted using the statistical software R. Descriptive statistics were used for demographic information. Wilcoxon-signed rank test was performed for within-group comparison comparing the baseline, end of treatment, and follow-up measures for the ESAS-17 and PROMIS-29. Statistical significance was set at 5%. Minimal clinically important difference (MCID) for ESAS is set 1-point reduction in the 11-point Likert scale for single symptoms and ≥ 3, ≥ 2, and ≥ 3 for physical, emotional, and total symptom distress scores [30]. Sample size calculation for a future RCT was conducted to detect for power of 0.8, using *t*-test for two independent groups.

## Results

**Fig. 1:**
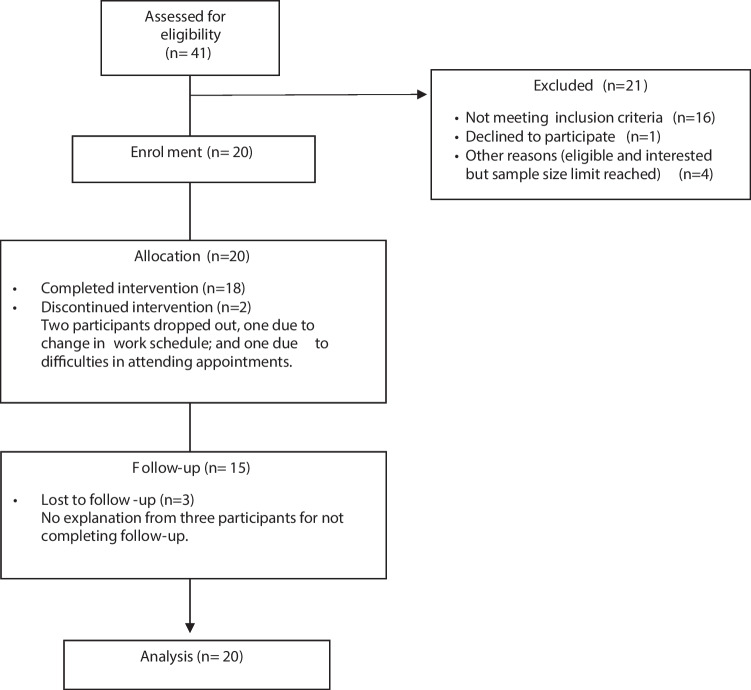
Participant flow chart

Forty-one women were assessed for eligibility, 20 women were not eligible, and 20 participants agreed to enroll in the study between 09/06/22 and 25/08/22 (Fig. 1). Two participants did not complete the intervention. Participants were on average 51.3 years old, with the majority diagnosed with estrogen receptor positive (90%) and HER-negative (85%) breast cancer (Table 1).

**Table 1:** Baseline characteristics

Characteristics	*N* (%)
Age	51.3 (range 29–72)
Location of cancer	Breast (100%)
Estrogen receptor positive (ER +)	18 (90%)
Progesterone receptor positive (PR +)	15 (75%)
Human epidermal growth factor receptor negative (HER −)	17 (85%)
Chemotherapy	20 (100%)
Radiotherapy	15 (75%)
Surgery	20 (100%)

### Feasibility

**Fig. 2:**
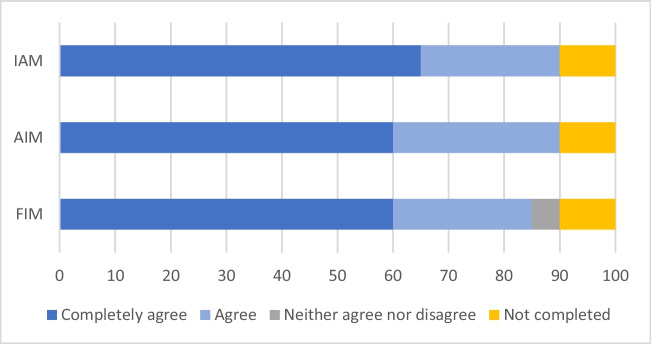
Feasibility of intervention measure, appropriateness of intervention measure, and intervention acceptability measure

The majority of participants found acupuncture to be feasible, acceptable, and appropriate for the management of this symptom cluster (Fig. 2). Seventeen participants (85%) agreed or completely agreed that the intervention was feasible. Eighteen participants (90%) agreed that the intervention was both acceptable and appropriate.

**Fig. 3:**
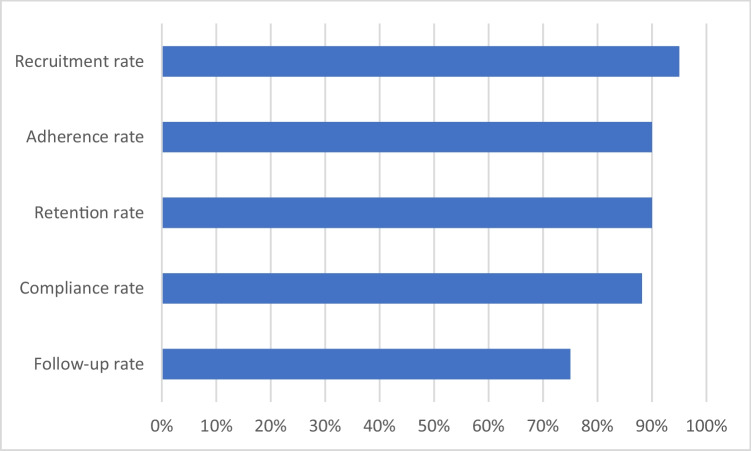
Recruitment, retention, adherence, compliance, and follow-up rates

Recruitment rate was at 95%, with 20 out of 21 eligible women enrolled, and retention rate was at 90%, with 18 participants completing the trial (Fig. 3). Eighteen participants (90%) attended at least six out of eight treatments, and compliance rate was 88% (141 out of 160 appointments attended). Eighteen participants (90%) completed the trial, and 15 participants (75%) completed follow-up.

### Edmonton Symptom Assessment Score (ESAS-17)

#### ***Symptom cluster score***

At baseline, the mean score for the sleep-fatigue-pain-numbness/tingling symptom cluster was 5.7 (Supplementary material 2). For individual symptoms, the mean score for sleep was 6.40 and 6.15 for fatigue, 5.30 for pain, and 5.10 for numbness/tingling (score 4–6: moderate).

**Fig. 4:**
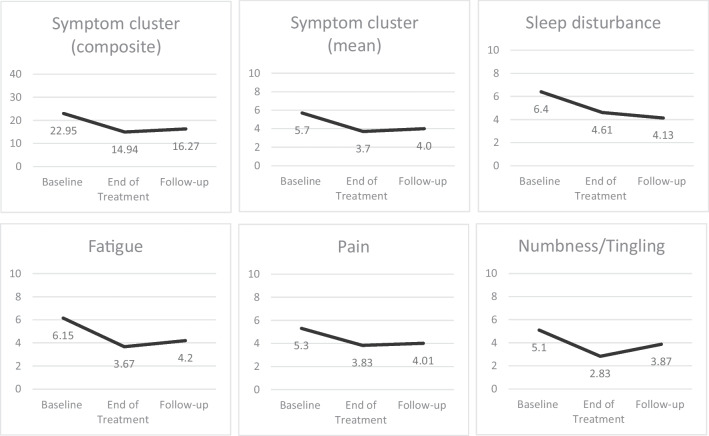
Change in ESAS mean scores. Note that all scores in the figure refer to the mean values

After completion of the acupuncture treatment, the symptom cluster mean score was reduced from 5.7 to 3.7 (mean difference 1.74, *p* < 0.001). The score remained lower at follow-up, with a mean difference of 1.45 (*p* = 0.02) from baseline to follow-up (Fig. 4).

#### Individual symptoms

For individual symptom scores, sleep disturbance reduced from 6.40 at baseline to 4.60 at the end of treatment and to 4.13 at follow-up; fatigue from 6.15 to 3.67 at the end of treatment and 4.2 at follow-up; and numbness/tingling from 5.1 to 2.83, which was marginally maintained (3.87) at follow-up. Pain scores reduced the least from 5.3 at baseline to 3.83 at the end of treatment and 4.01 at follow-up (Fig. 4). All reductions in the individual symptoms related to the symptom cluster were clinically significant.

At baseline, sleep disturbance and fatigue were the most prevalent clinically significant symptoms (≥ 4 ESAS), experienced by 95% and 85% of participants, respectively.

#### Distress scores

Decreases in mean ESAS group scores were clinically and statistically significant from baseline to end of treatment. Decreases in mean ESAS group scores were also clinically and statistically significant from baseline to end of treatment. Physical distress was reduced from 21.25 to 14.22 (mean difference 5.61, *p* = 0.04), emotional distress from 9.15 to 5.5 (mean difference 3.17, *p* = 0.03), and global distress from 35.15 to 23.83 (mean difference 9.33, *p* = 0.02) (Supplementary material 2).

#### Changes in other symptoms

For other ESAS symptoms not within the pain-fatigue-sleep-numbness symptom cluster, there was a statistically and clinically significant improvement for anxiety at end of treatment (1.9, *p* = 0.02) and follow-up (2, *p* = 0.01), and for depression at end of treatment (1.2, *p* < 0.05).

### Traditional Chinese Medicine syndromes

The overall symptom cluster and individual symptoms were examined for correlations between TCM syndromes identified using the SOHQ-50 (Table 2). See Supplementary material 3 for the explanations on TCM syndrome terminology.

**Table 2:** Correlation between symptom cluster and TCM syndromes

	TCM syndrome	Correlation (*r*)
Symptom cluster	*Spleen qi deficiency*	0.69
	*Heart fire*	0.57
Pain	*Spleen qi deficiency*	0.43
Fatigue	*Spleen qi deficiency*	0.69
Sleep disturbance	*Heart fire*	0.65
Numbness/tingling	*Stomach fire*	0.21

*Moderate correlation r* = *0.4–0.7*

At baseline, the symptom cluster of pain-fatigue-sleep disturbance-numbness/tingling was most closely associated with *Spleen qi deficiency* (ICD-11 code SF70) (*r* = 0.69) and *Heart fire* (SF69) (*r* = 0.57). For single symptoms, fatigue was most closely associated with *Spleen qi deficiency* (*r* = 0.69), pain with *Spleen qi deficiency* (*r* = 0.43), numbness/tingling with *Stomach fire* (SF7F) (*r* = 0.21), and sleep disturbance with *Heart fire* (*r* = 0.65). For group scores, physical distress was strongly associated with *Spleen qi deficiency* (*r* = 0.71), emotional distress with *Damp retention* (SF13) (*r* = 0.72), and global distress with *Damp retention* (*r* = 0.75)*.*

### Correlation between symptoms

There was a moderate correlation between fatigue and sleep disturbance (*r* = 0.65) and a mild correlation between sleep disturbance and pain (*r* = 0.39).

### PROMIS-29

Using the PROMIS-29, fatigue was significantly reduced at end of treatment (*p* = 0.01) and follow-up (*p* = 0.01) (Supplementary material 2). Social activity and pain interference also showed statistically significant reductions at follow-up (*p* = 0.004, *p* < 0.05, respectively). Other changes, including the PROPr score, were not statistically significant.

### Sample size calculation

A sample size calculation was performed for 80% power and statistical significance of 0.05. Clinical difference was estimated as 1-point reduction in symptom cluster mean score, based on MCID of the individual symptoms on the ESAS [29]. Standard deviation of 1.45 was calculated for an effect size of 0.69. A sample size of 68 participants was calculated, with 34 participants in each control and intervention groups.

### Adverse events

No adverse events were reported.

## Discussion

### Interpretation of findings

Our study demonstrated that acupuncture for breast cancer survivors with the pain-fatigue-sleep disturbance-numbness/tingling symptom cluster is a feasible intervention, with high retention and adherence rates. Moreover, most participants perceived acupuncture to be acceptable, appropriate, and feasible for their ongoing cancer care. Mean symptom scores for the cluster at completion of intervention and at follow-up showed significant improved compared baseline level. This signaled potential benefit of acupuncture for reducing the burden of this cluster and maintaining this benefit after cessation of treatment. The TCM syndrome described as “*Spleen qi deficiency*” was most closely associated with this symptom cluster, and may be a target for future acupuncture protocols. Further studies are needed to assess effectiveness, dosage, duration, and a longer follow-up period. Mechanisms of effectiveness could also be explored in a future study.

### Strengths

The integration of acupuncture into oncology settings is hampered by insufficient early phase research that provide preliminary data for efficacy trials, sub-optimal treatment protocols that are over-simplified and do not reflect clinical practice, and narrow assessment of outcomes which focus on single symptoms rather than TCM syndromes [20, 21, 38]. The strengths of our study aimed to address these issues by providing the preliminary data for an efficacy trial, and using a symptom cluster as an outcome measure [21].

Furthermore, our acupuncture intervention reflected clinical practice by being pragmatically designed, where acupuncturists conducted a typical syndrome diagnosis and selected acupuncture points according to this diagnosis. Model validity in complex interventions such as acupuncture is critical to ensure accurate findings in a clinical trial — when model validity is low this may result in “false negative” findings due to suboptimal treatment [39].

### Previous research

To our knowledge, this trial is the first of its kind to examine the feasibility of acupuncture in the management of a symptom cluster in people with cancer. Previous trials have largely focused on acupuncture for a single symptom [40]. A small number of trials have examined multiple symptoms, but not a pre-defined symptom cluster, where symptoms were selected based on the characteristics of the patient group, a core symptom and its associated symptoms, or by the prevalence or severity of the symptoms [41, 42]. In this way, these trials examine the symptoms independent of each other, rather than as a pre-defined cluster of inter-related symptoms. Symptom cluster research using acupuncture is emerging, and the findings of a recently published research protocol focusing on a symptom cluster involving cognitive function will provide additonal insights [43].

Our findings on TCM syndromes are consistent with previous studies which have associated breast cancer with qi and xie (blood) deficiency [44]. Future studies may be guided by these findings in designing acupuncture treatment protocols for this cohort.

### Limitations

Our study has several limitations. First, the study was a single cohort in a single setting, without blinding of participants or practitioners, reducing external validity, and increasing risk of bias. Secondly, the acupuncture intervention was completely individualized and determined according to the expertise and clinical reasoning of the acupuncturist on the day (which may not be based on the SOHQ-50 taken at baseline). While this pragmatic approach reflects the clinical practice of acupuncture, it limits the reproducibility and generalizability of the intervention. Thirdly, there is no gold standard tool to diagnose TCM syndromes, and the SOHQ-50 may present a limited analysis on the complexities of TCM diagnosis.

Finally, there is no validated, standardized outcome for measuring this symptom cluster. Symptom cluster research is in early stages [10, 45] and the MCID for this pain-fatigue-sleep disturbance-numbness/tingling symptom cluster has not been previously investigated. In this study, a “symptom cluster score” was calculated by the mean of pain, fatigue, sleep disturbance, and numbness/tingling on the ESAS-17, to allow for comparison with the MCID of other single symptoms.

### Future directions

The findings from this trial are consistent with other research on the effectiveness of acupuncture treatment for single and multiple symptoms in people with cancer [26, 41]. Building on these findings, a RCT examining the effectiveness of acupuncture for the management of multiple-concurrent symptoms in this pain-fatigue-sleep disturbance-numbness/tingling cluster is warranted.

## Conclusion

This trial demonstrated that acupuncture was a feasible, acceptable, and appropriate treatment for the management of the pain-fatigue-sleep disturbance-numbness/tingling symptom cluster in breast cancer survivors. The results signal potential beneficial effects of acupuncture for the fatigue-sleep-pain-numbness/tingling symptom cluster. Future research is needed to determine if these benefits are achieved in a randomized controlled trial.

### Supplementary Information

Below is the link to the electronic supplementary material.

Supplementary file1 (DOCX 26 KB)

Supplementary file2 (DOCX 29 KB)

Supplementary file3 (DOCX 21 KB)

## Data Availability

Data that support the findings of this study is available on request from the corresponding author.
