# Supplementary material for: Acupuncture for the treatment of the pain-fatigue-sleep disturbance-numbness/tingling symptom cluster in breast cancer survivors: a feasibility trial
Source: Support Care Cancer. 2024 May 7;32(6):332. doi: 10.1007/s00520-024-08529-9 (PMC11076364; doi:10.1007/s00520-024-08529-9)
Supplement: Supplementary file 1 — Supplementary file1 (DOCX 26 KB) [file 520_2024_8529_MOESM1_ESM.docx]

**Acupuncture for the treatment of the pain-fatigue-sleep disturbance-numbness/tingling symptom cluster in breast cancer survivors: a pilot feasibility trial**

Supportive Care in Cancer

Ki Kyung Kwon^1, 2^, Judith Lacey^1, 2,3^, Kim Kerin-Ayres^2^, Gillian Heller^2,3^ & Suzanne Grant^1,2^

1. NICM Health Research Institute, Western Sydney University, Sydney, Australia
2. Chris O’Brien Lifehouse Hospital, Camperdown, Australia
3. University of Sydney, Camperdown, Australia

Email: ki.kwon@lh.org.au

*Supplement 1.1: STRICTA checklist*

| Item | Detail | Response |
| --- | --- | --- |
| 1. Acupuncture rationale | 1a) Style of acupuncture | Traditional Chinese Medicine |
|  | 1b) reasoning for treatment provided, based on historical context, literature sources, and/or consensus methods, with references where available | Clinical reasoning using on TCM diagnosis based on Deadman et al. (2007) |
|  | 1c) Extent to which treatment was varied | There was no standardized points. Each treatment was different based on the patient interview and examination on the day |
| 1. Details of needling | 2a) Number of needle insertions per subject per session | Varied each session for each patient based on the patient interview and examination on the day, between 5-10 points |
|  | 2b) Names (or location) of points used | Points varied for each session and each patient based on the patient interview and examination on the day. Points used were based on Deadman et al., (2007) |
|  | 2c) Depth of insertion, based on a specified unit of measurement, or on a particular tissue level | Depth of insertion varied from point to point, based on the recommendations by Deadman et al. (2007) |
|  | 2d) Response sought | De-qi sensation |
|  | 2e) Needle stimulation | EA where appropriate  Varied point to point, based on Deadman et al. (2007) |
|  | 2f) Needle retention time | 20-30 minutes |
|  | 2g) Needle type | 0.16x0.20 to 0.25x0.40, Vinco brand |
| 1. Treatment regimen | 3a) Number of treatment sessions | 8 sessions |
|  | 3b) Frequency and duration of treatment sessions | 2 per week for first two weeks, then 1 per week for last four weeks, 40-60 min per session |
| 1. Other components of treatment | 4a) Details of other interventions administered to the acupuncture group | NA |
|  | 4b) Setting and context of treatment, including instructions to practitioners, and information and explanation to patients | Single arm feasibility trial conducted at COBLH. Practitioners were instructed to approach the treatment as they would normally, outside of the trial. |
| 1. Practitioner background | Description of participating acupuncturists (qualification, professional affiliation, years in acupuncture practice, other relevant experience) | Two acupuncturists who are registered with AHPRA, and 5+ years in working with patients with cancer administered the treatments. |
| 1. Control or comparator interventions | 6a) rationale for the control or comparator in the context of the research question, with sources that justify this choice | No control or comparator group was employed in this trial as the primary aim was to test feasibility, not treatment efficacy |
|  | 6b) Precise description of the control or comparator | NA |

*Supplement 1.2: CONSORT checklist*

|  |  | Reporting Item | Page Number |
| --- | --- | --- | --- |
| **Title and Abstract** |  |  |  |
| Title | [#1a](https://www.goodreports.org/reporting-checklists/consort/info/#1a) | Identification as a randomized trial in the title. | n/a (single arm feasibility trial) |
| Abstract | [#1b](https://www.goodreports.org/reporting-checklists/consort/info/#1b) | Structured summary of trial design, methods, results, and conclusions | 2 |
| **Introduction** |  |  |  |
| Background and objectives | [#2a](https://www.goodreports.org/reporting-checklists/consort/info/#2a) | Scientific background and explanation of rationale | 3 |
| Background and objectives | [#2b](https://www.goodreports.org/reporting-checklists/consort/info/#2b) | Specific objectives or hypothesis | 4 |
| **Methods** |  |  |  |
| Trial design | [#3a](https://www.goodreports.org/reporting-checklists/consort/info/#3a) | Description of trial design (such as parallel, factorial) including allocation ratio. | 5 |
| Trial design | [#3b](https://www.goodreports.org/reporting-checklists/consort/info/#3b) | Important changes to methods after trial commencement (such as eligibility criteria), with reasons | n/a |
| Participants | [#4a](https://www.goodreports.org/reporting-checklists/consort/info/#4a) | Eligibility criteria for participants | 5 |
| Participants | [#4b](https://www.goodreports.org/reporting-checklists/consort/info/#4b) | Settings and locations where the data were collected | 5 |
| Interventions | [#5](https://www.goodreports.org/reporting-checklists/consort/info/#5) | The experimental and control interventions for each group with sufficient details to allow replication, including how and when they were actually administered | 6 |
| Outcomes | [#6a](https://www.goodreports.org/reporting-checklists/consort/info/#6a) | Completely defined prespecified primary and secondary outcome measures, including how and when they were assessed | 6-7 |
| Sample size | [#7a](https://www.goodreports.org/reporting-checklists/consort/info/#7a) | How sample size was determined. | 7 |
| Sample size | [#7b](https://www.goodreports.org/reporting-checklists/consort/info/#7b) | When applicable, explanation of any interim analyses and stopping guidelines | n/a |
| Randomization - Sequence generation | [#8a](https://www.goodreports.org/reporting-checklists/consort/info/#8a) | Method used to generate the random allocation sequence. |  |
| n/a |  |  |  |
| Randomization - Sequence generation | [#8b](https://www.goodreports.org/reporting-checklists/consort/info/#8b) | Type of randomization; details of any restriction (such as blocking and block size) |  |
| n/a |  |  |  |
| Randomization - Allocation concealment mechanism | [#9](https://www.goodreports.org/reporting-checklists/consort/info/#9) | Mechanism used to implement the random allocation sequence (such as sequentially numbered containers), describing any steps taken to conceal the sequence until interventions were assigned | n/a |
| Randomization - Implementation | [#10](https://www.goodreports.org/reporting-checklists/consort/info/#10) | Who generated the allocation sequence, who enrolled participants, and who assigned participants to interventions | n/a |
| Blinding | [#11a](https://www.goodreports.org/reporting-checklists/consort/info/#11a) | If done, who was blinded after assignment to interventions (for example, participants, care providers, those assessing outcomes) and how. | n/a |
| Blinding | [#11b](https://www.goodreports.org/reporting-checklists/consort/info/#11b) | If relevant, description of the similarity of interventions | n/a |
| Statistical methods | [#12a](https://www.goodreports.org/reporting-checklists/consort/info/#12a) | Statistical methods used to compare groups for primary and secondary outcomes | 8 |
| Statistical methods | [#12b](https://www.goodreports.org/reporting-checklists/consort/info/#12b) | Methods for additional analyses, such as subgroup analyses and adjusted analyses | n/a |
| Outcomes | [#6b](https://www.goodreports.org/reporting-checklists/consort/info/#6b) | Any changes to trial outcomes after the trial commenced, with reasons | n/a |
| **Results** |  |  |  |
| Participant flow diagram (strongly recommended) | [#13a](https://www.goodreports.org/reporting-checklists/consort/info/#13a) | For each group, the numbers of participants who were randomly assigned, received intended treatment, and were analysed for the primary outcome | 8 |
| Participant flow | [#13b](https://www.goodreports.org/reporting-checklists/consort/info/#13b) | For each group, losses and exclusions after randomization, together with reason | 8 |
| Recruitment | [#14a](https://www.goodreports.org/reporting-checklists/consort/info/#14a) | Dates defining the periods of recruitment and follow-up | 9 |
| Recruitment | [#14b](https://www.goodreports.org/reporting-checklists/consort/info/#14b) | Why the trial ended or was stopped | n/a |
| Baseline data | [#15](https://www.goodreports.org/reporting-checklists/consort/info/#15) | A table showing baseline demographic and clinical characteristics for each group | 9 |
| Numbers analysed | [#16](https://www.goodreports.org/reporting-checklists/consort/info/#16) | For each group, number of participants (denominator) included in each analysis and whether the analysis was by original assigned groups | 8 |
| Outcomes and estimation | [#17a](https://www.goodreports.org/reporting-checklists/consort/info/#17a) | For each primary and secondary outcome, results for each group, and the estimated effect size and its precision (such as 95% confidence interval) | 9-12 |
| Outcomes and estimation | [#17b](https://www.goodreports.org/reporting-checklists/consort/info/#17b) | For binary outcomes, presentation of both absolute and relative effect sizes is recommended | n/a |
| Ancillary analyses | [#18](https://www.goodreports.org/reporting-checklists/consort/info/#18) | Results of any other analyses performed, including subgroup analyses and adjusted analyses, distinguishing pre-specified from exploratory | n/a |
| Harms | [#19](https://www.goodreports.org/reporting-checklists/consort/info/#19) | All important harms or unintended effects in each group (For specific guidance see CONSORT for harms) | 12 |
| **Discussion** |  |  |  |
| Limitations | [#20](https://www.goodreports.org/reporting-checklists/consort/info/#20) | Trial limitations, addressing sources of potential bias, imprecision, and, if relevant, multiplicity of analyses | 14 |
| Interpretation | [#22](https://www.goodreports.org/reporting-checklists/consort/info/#22) | Interpretation consistent with results, balancing benefits and harms, and considering other relevant evidence | 13 |
| Registration | [#23](https://www.goodreports.org/reporting-checklists/consort/info/#23) | Registration number and name of trial registry | 9 |
| Generalisability | [#21](https://www.goodreports.org/reporting-checklists/consort/info/#21) | Generalisability (external validity, applicability) of the trial findings | 13 |
| **Other information** |  |  |  |
| Interpretation | [#22](https://www.goodreports.org/reporting-checklists/consort/info/#22) | Interpretation consistent with results, balancing benefits and harms, and considering other relevant evidence | 13 |
| Registration | [#23](https://www.goodreports.org/reporting-checklists/consort/info/#23) | Registration number and name of trial registry | 9 |
| Protocol | [#24](https://www.goodreports.org/reporting-checklists/consort/info/#24) | Where the full trial protocol can be accessed, if available | n/a |
| Funding | [#25](https://www.goodreports.org/reporting-checklists/consort/info/#25) | Sources of funding and other support (such as supply of drugs), role of funders | n/a |

Notes:

- 1a: n/a (single arm feasibility trial) The CONSORT checklist is distributed under the terms of the Creative Commons Attribution License CC-BY. This checklist was completed on 04. August 2023 using <https://www.goodreports.org/>, a tool made by the [EQUATOR Network](https://www.equator-network.org) in collaboration with [Penelope.ai](https://www.penelope.ai)
