# Supplementary material for: Acupuncture for the treatment of the pain-fatigue-sleep disturbance-numbness/tingling symptom cluster in breast cancer survivors: a feasibility trial
Source: Support Care Cancer. 2024 May 7;32(6):332. doi: 10.1007/s00520-024-08529-9 (PMC11076364; doi:10.1007/s00520-024-08529-9)
Supplement: Supplementary file 2 — Supplementary file2 (DOCX 29 KB) [file 520_2024_8529_MOESM2_ESM.docx]

**Acupuncture for the treatment of the pain-fatigue-sleep disturbance-numbness/tingling symptom cluster in breast cancer survivors: a pilot feasibility trial**

Supportive Care in Cancer

Ki Kyung Kwon^1, 2^, Judith Lacey^1, 2,3^, Kim Kerin-Ayres^2^, Gillian Heller^2,3^ & Suzanne Grant^1,2^

1. NICM Health Research Institute, Western Sydney University, Sydney, Australia
2. Chris O’Brien Lifehouse Hospital, Camperdown, Australia
3. University of Sydney, Camperdown, Australia

Email: ki.kwon@lh.org.au

*Supplementary Table 2.1: Time points for outcome measures*

| **Outcome Measures** | **Enrolment** | **Week 1-6** | **Week 6** | **Follow-up** |
| --- | --- | --- | --- | --- |
| Edmonton Symptom Assessment Scale-17 | ✓ |  | ✓ | ✓ |
| Patient Reported Outcome Measure Information System-29 | ✓ |  | ✓ | ✓ |
| Sub Health Status Questionnaire-50 | ✓ |  |  |  |
| Acceptability of Intervention Measure, Feasibility of Intervention Measure, Intervention Appropriate Measure |  |  | ✓ |  |
| Credibility and Expectancy Questionnaire | ✓ |  | ✓ |  |
| Adverse Event Assessment |  | ✓ |  |  |

|  | Baseline | | End-point  (6 weeks) | | Follow-up  (8 weeks) | | Baseline –  End-point | | Baseline –  Follow-up | |
| --- | --- | --- | --- | --- | --- | --- | --- | --- | --- | --- |
|  | Prevalence (%) | Mean  (sd) | Prevalence (%) | Mean  (sd) | Prevalence (%) | Mean  (sd) | Mean  Difference  (sd) | p | Mean  Difference  (sd) | p |
| Pain | 70 | 5.3 (2.6) | 39 | 3.83 (2.5) | 50 | 4.07 (2.5) | 1.22 (2.5) | 0.077 | 1.33  (2.0) | 0.191 |
| Fatigue | 85 | 6.15 (2.6) | 44 | 3.67 (2.2) | 50 | 4.2 (2.6) | 2.17* (2.3) | *0.004* | 1.33*  (2.2) | *0.04* |
| Sleep | 95 | 6.4 (2.3) | 56 | 4.61 (2.8) | 45 | 4.13 (2.7) | 1.5* (3.1) | *0.037* | 1.93*  (2.3) | *0.014* |
| Numbness | 70 | 5.1 (2.7) | 28 | 2.83 (2.6) | 40 | 3.87 (2.8) | 2.01* (1.8) | *0.012* | 1.2  (2.3) | 0.215 |
| Symptom cluster (composite) | - | 22.95 (6.1) | - | 14.94 (6.6) | - | 16.27 (7.9) | 6.94* (5.8) | *0.00008* | 5.8*  (5.7) | *0.018* |
| Symptom cluster (mean) | - | 5.7 (1.5) | - | 3.7 (1.7) | - | 4.1 (2.0) | 1.74* (1.5) | *0.0008* | 1.45*  (1.4) | *0.018* |
| Nausea | 15 | 1.5 (2.0) | 6 | 0.78 (1.5) | 0 | 0.73 (1.1) | 0.44 (1.9) | 0.215 | 0.4  (1.9) | 0.234 |
| Anxiety | 75 | 5.2 (2.9) | 39 | 3.11 (2.1) | 30 | 2.87 (1.9) | 1.94* (2.3) | *0.018* | 2*  (2.0) | *0.011* |
| Depression | 60 | 3.95 (2.8) | 28 | 2.39 (2.2) | 20 | 2.13 (1.8) | 1.22 (1.7) | 0.091 | 1.2*  (1.8) | *0.047* |
| Drowsiness | 40 | 3.1 (3.0) | 22 | 2 (2.1) | 20 | 1.93 (2.1) | 1.11  (2.2) | 0.308 | 1.13  (1.3) | 0.215 |
| Appetite | 40 | 2.8 (2.8) | 39 | 2.44 (2.8) | 40 | 3.13 (3.0) | 0  (3.4) | 0.686 | -0.93  (3.9) | 0.865 |
| Short breath | 35 | 2.4 (2.3) | 17 | 1.5 (1.9) | 15 | 1.4 (1.9) | 0.67  (1.8) | 0.278 | 0.67  (1.6) | 0.213 |
| Wellbeing | 75 | 4.75 (1.5) | 50 | 4.11 (2.6) | 55 | 4.53 (1.8) | 0.56  (3.5) | 0.393 | -0.13  (2.9) | 0.685 |
| Financial | 65 | 4.6 (3.4) | 39 | 3.39 (3.4) | 35 | 3.93 (3.4) | 1.33  (2.2) | 0.316 | 0.533  (1.6) | 0.591 |
| Spiritual | 30 | 2.1 (2.7) | 11 | 1.72 (2.1) | 5 | 1.47 (2.0) | 0.61  (2.2) | 1 | 0.4  (1.7) | 0.602 |
| Hot flashes | 50 | 3.85 (3.1) | 33 | 2.83 (2.0) | 45 | 3.33 (2.4) | 0.94  (3.1) | 0.353 | 0.13  (2.6) | 0.813 |
| Dry mouth | 30 | 2.35 (2.0) | 28 | 2.44 (2.8) | 20 | 1.73 (1.9) | -0.44  (2.2) | 0.893 | -0.13  (1.5) | 0.506 |
| Memory | 45 | 3.5 (2.5) | 56 | 3.22 (2.1) | 20 | 4.07 (1.8) | 0.39  (1.7) | 0.812 | 0.73  (1.3) | 0.25 |
| Other | 35 | 2.1 (3.3) | 11 | 1.67 (2.1) | 10 | 0.91 (1.6) | 2.11  (3.4) | 0.922 | 1.45  (2.8) | 0.221 |
| Physical distress | - | 21.25 (10.0) | - | 14.22 (9.3) | - | 15.47 (9.4) | 5.61*  (8.8) | *0.042* | 3.93  (7.7) | 0.204 |
| Emotional distress | - | 9.15 (5.5) | - | 5.5 (4.0) | - | 5 (3.4) | 3.17*  (3.8) | *0.032* | 3.2*  (3.5) | *0.023* |
| Global distress | - | 35.15 (15.7) | - | 23.83 (12.5) | - | 25 (11.8) | 9.33*  (12.3) | *0.015* | 7*  (10.5) | *0.029* |

*Supplementary Table 2.2:* *Change in ESAS-17 means over time*

*Supplementary Table 2.3.* *Mean within group difference for PROMIS-29*

|  | Baseline – End-point | | Baseline – Follow-up | |
| --- | --- | --- | --- | --- |
|  | Mean difference | P-value | Mean difference | P-value |
| Physical activity | 1.31 | 0.37 | 2.85 | 0.14 |
| Anxiety | 2.94 | 0.23 | 2.73 | 0.08 |
| Depression | 2.69 | 0.23 | 1.51 | 0.35 |
| Fatigue | 6.28* | 0.006 | 6.71* | 0.009 |
| Sleep | 0.64 | 0.48 | 0.63 | 0.44 |
| Social activity | 3.94 | 0.09 | 5.83* | 0.004 |
| Pain interference | 4.67 | 0.08 | 6.38* | 0.05 |
| PROSPr | -0.005 | 0.76 | 0.0156 | 0.53 |
