# Supplementary material for: Acupuncture for the treatment of the pain-fatigue-sleep disturbance-numbness/tingling symptom cluster in breast cancer survivors: a feasibility trial
Source: Support Care Cancer. 2024 May 7;32(6):332. doi: 10.1007/s00520-024-08529-9 (PMC11076364; doi:10.1007/s00520-024-08529-9)
Supplement: Supplementary file 3 — Supplementary file3 (DOCX 21 KB) [file 520_2024_8529_MOESM3_ESM.docx]

**Acupuncture for the treatment of the pain-fatigue-sleep disturbance-numbness/tingling symptom cluster in breast cancer survivors: a pilot feasibility trial**

Supportive Care in Cancer

Ki Kyung Kwon^1, 2^, Judith Lacey^1, 2,3^, Kim Kerin-Ayres^2^, Gillian Heller^2,3^ & Suzanne Grant^1,2^

1. NICM Health Research Institute, Western Sydney University, Sydney, Australia
2. Chris O’Brien Lifehouse Hospital, Camperdown, Australia
3. University of Sydney, Camperdown, Australia

Email: ki.kwon@lh.org.au

Supplementary material 3: Traditional Chinese Medicine (TCM) terminology

Clinical practice of acupuncture is designed to treat TCM syndromes. TCM syndromes consider multiple, concomitant symptoms that have a theoretical basis for shared relationships or mechanisms. In this way, the conceptualization of TCM syndromes may be considered in relation to symptom cluster management.

Terminology:

Qi is the base form of energy used in the regular, healthy functioning of our body (Hong, 2013; Hsu et al., 2018)

Spleen functional system is considered to be responsible for transforming the food and drinks we ingest into the form of qi (energy) we use in our bodies and distributing that qi to the rest of the body. This can be considered in relation to the digestive system.

In the distribution of qi, there are two main considerations. One is a deficiency, where there is a lack, and one is an excess, where there is stagnation or congestion.

Spleen qi deficiency would therefore be a state where the digestive function is not functioning optimally to transform and distribute the energy that is required in our body. This results in a state of hypoactivity of body systems, manifesting as slower digestion, nausea, low energy, depression, shortness of breath, poor circulation, and weakness. While not all the symptoms seem directly related to the digestive function, they are considered the branch symptoms arising from the root cause (Ching, 2016; Jiang et al., 2012).

Heart is considered to house the *shen* in TCM theory. *Shen* can be roughly conceptualized in terms of the mind, spirit, emotions (Mole, 2014).

Fire is a term for describing a strong excess. The excess is usually a build up of congestion due to a stagnation in the distribution of the qi.

In context of *Heart fire*, this would mean that there is congestion in the system where the mind, spirit, emotions are governed. Consider the manifesting symptoms as disturbed sleep, mood swings, irritation, anxiety, and other psychological distress, in a general state of hyperactivity (Jiang et al., 2012).

References

Ching, N. (2016). *The fundamentals of acupuncture*. Singing Dragon.

Hong, H. (2013). *Acupuncture: Theories and evidence*. World Scientific.

Hsu, E. S., Wu, I., & Lai, B. (2018). Acupuncture. In *Essentials of pain medicine* (pp. 545-550. e541). Elsevier.

Jiang, M., Lu, C., Zhang, C., Yang, J., Tan, Y., Lu, A., & Chan, K. J. J. o. e. (2012). Syndrome differentiation in modern research of traditional Chinese medicine. *140*(3), 634-642.

Mole, P. (2014). *Acupuncture for Body, Mind and Spirit*. Singing Dragon.
